# Supplementary material for: Isoform- and ligand-specific modulation of the adhesion GPCR ADGRL3/Latrophilin3 by a synthetic binder
Source: Nat Commun. 2023 Feb 6;14:635. doi: 10.1038/s41467-023-36312-7 (PMC9902482; doi:10.1038/s41467-023-36312-7)
Supplement: Supplementary file 1 — Supplementary Information [file 41467_2023_36312_MOESM1_ESM.pdf]

Supplementary Figs. 1-10

for

Isoform- and ligand-specific modulation of the adhesion GPCR ADGRL3/Latrophilin3 by a synthetic binder

Szymon P. Kordon[1,2,3], Przemysław Dutka[1,4], Justyna M. Adamska[1,2,3], Sumit J. Bandekar[1,2,3], Katherine Leon[1,2,3], Satchal K. Erramilli[1], Brock Adams[1,2,3], Jingxian Li[1,2,3], Anthony A. Kossiakoff[1,3], Demet Araç[1,2,3,*]

1 Department of Biochemistry and Molecular Biology, The University of Chicago, Chicago, IL, USA

2 Neuroscience Institute, The University of Chicago, Chicago, IL, USA

3 Institute for Biophysical Dynamics, University of Chicago, Chicago, IL, USA

4 Present address: Division of Chemistry and Chemical Engineering and Division of Biology and Biological Engineering, California Institute of Technology, Pasadena, CA, USA

* Correspondence: arac@uchicago.edu

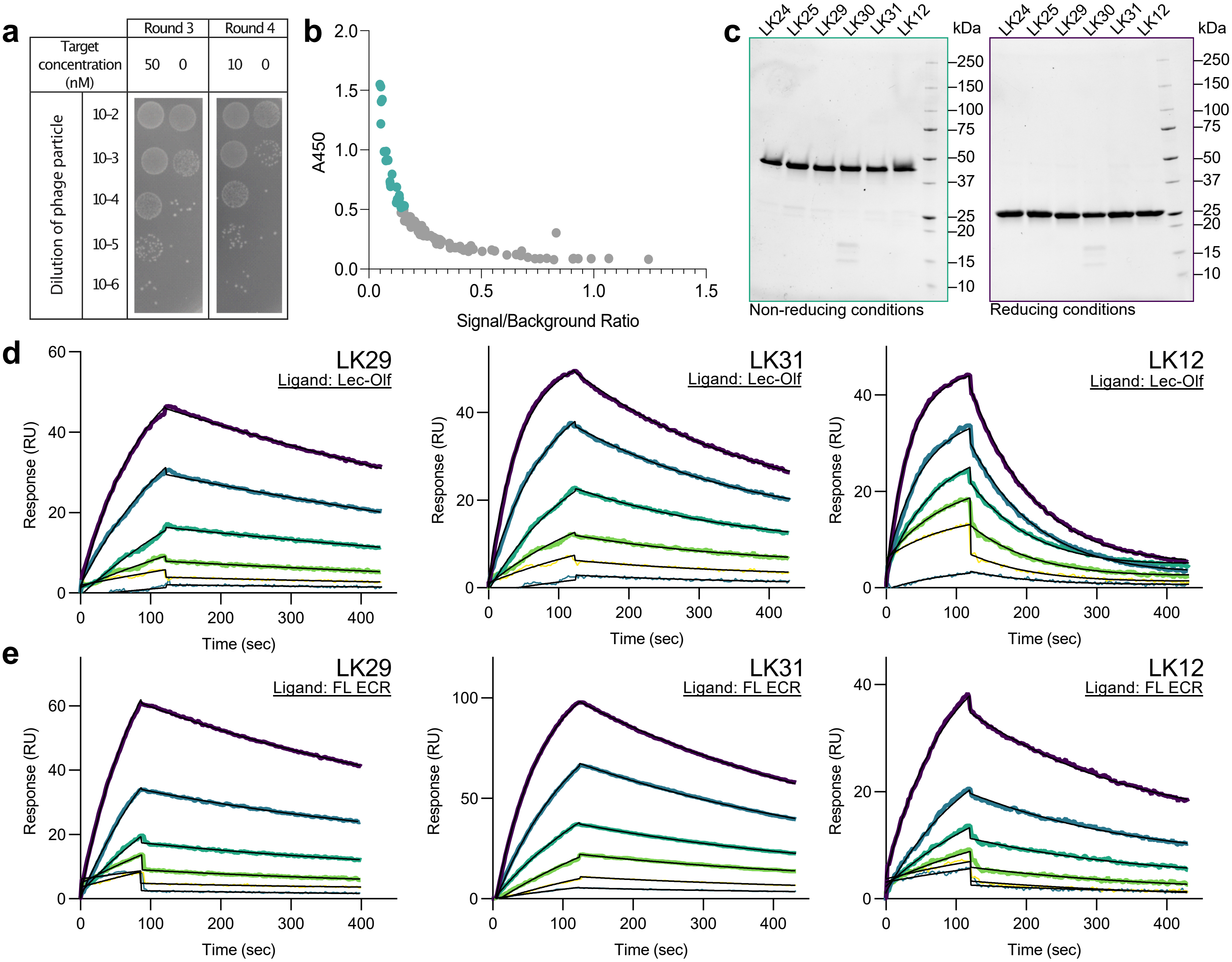

f

sAB ID	ADGRL3					
	Lec/Olf			Full ECR		
	k_{on} ($M^{-1}s^{-1}$)	k_{off} (s^{-1})	K_D (nM)	k_{on} ($M^{-1}s^{-1}$)	k_{off} (s^{-1})	K_D (nM)
LK24	NB			NB		
LK25	NB			Equilibrium		56.8
LK29	4.1 x 10^5	1.0 x 10^{-3}	2.5	3.8 x 10^5	1.5 x 10^{-3}	4.1
**LK30**	**1.7 x 10^6**	**4.9 x 10^{-3}**	**5.4**	**1.4 x 10^6**	**5.8 x 10^{-3}**	**4.2**
LK31	8.2 x 10^5	2.9 x 10^{-3}	3.6	5.3 x 10^5	2.0 x 10^{-3}	3.8
**LK12**	**8.0 x 10^5**	**9.9 x 10^{-3}**	**12.5**	**3.8 x 10^5**	**3.2 x 10^{-3}**	**8.3**

Affinity of sAB LK25 was determined by ELISA, because fitting of the data from SPR was not possible.
NB = no binding detected.

g

LK30	
**CDR**	**Sequence**
**L3**	SNTELV
**H1**	IYYSSI
**H2**	YISPYSGSTS
**H3**	YKYGQGHMGAF

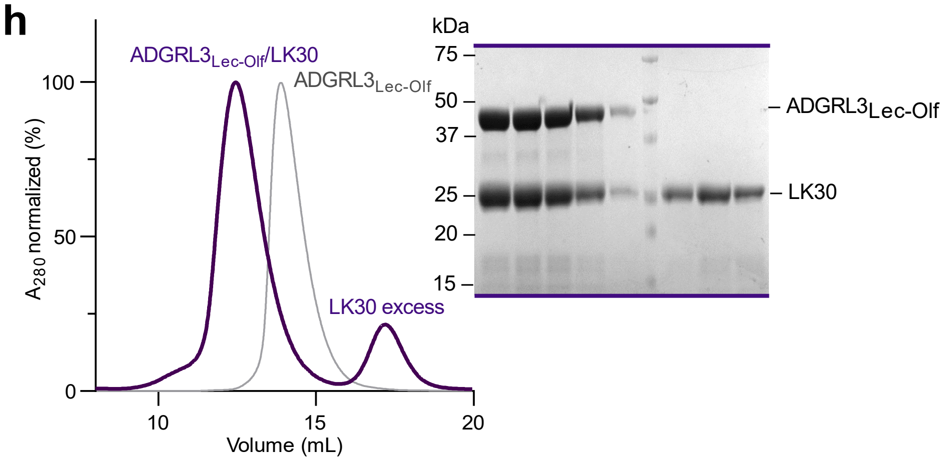

Supplementary Fig. 1: Development and characterization of sABs against ADGRL3.

a Enrichment of the target-specific binders after third and fourth round of antibody selection for full ECR of ADGRL3. **b** Binders obtained after four rounds of phage display antibody selection were screened by a single-point phage ELISA. Clones marked in cyan were further characterized. **c** SDS-PAGE analysis of sABs of interest in non-reducing (left) and reducing (right) conditions, showing the disruption of disulfide bonds by reducing agent in the loading buffer. Images representative of at least three independent experiments. **d** and **e** Surface plasmon resonance measurements of sABs LK29, LK31 and LK12 binding to the purified Lec-Olf fragment of ADGRL3 (**d**) and full-legth ECR of ADGRL3 (**e**). Each sAB concentration is shown in a different color trace. Within each plot, the multiconcentration global fit line is shown in black. In order from highest to lowest, the concentrations of analyte used were 25, 12.5, 6.25, 3.125, 1.563 and 0.781 nM. **f** Kinetic values of unique sABs developed against ECR of ADGRL3. **g** Sequences of LK30 CDRs. **h** SEC profiles and SDS-PAGE analyses of the LK30 complex with Lec-Olf fragment of ADGRL3. Results representative of two independent experiments.

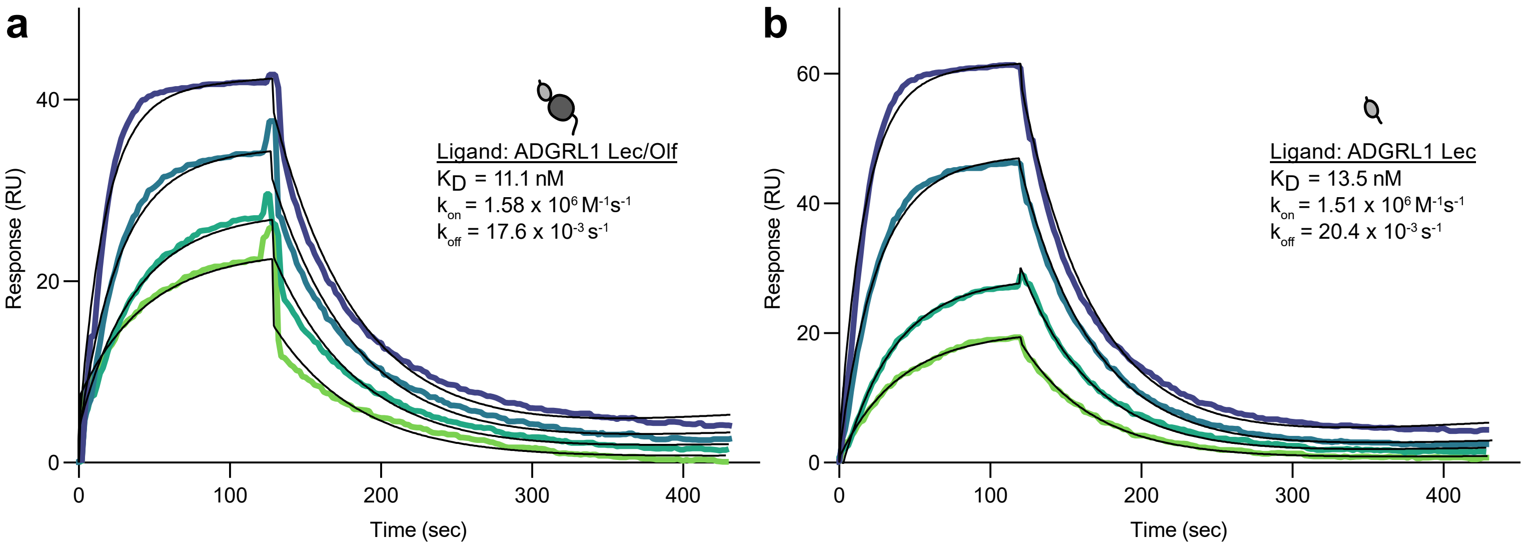

Supplementary Fig. 2: Kinetic characterization of sAB LK30 against ADGRL1 constructs.
Surface plasmon resonance measurements of the LK30 binding to purified ADGRL1 Lec/Olf fragment (**a**) and Lec domain (**b**). Each sAB concentration is shown in a different color trace. Within each plot, the multiconcentration global fit line is shown in black. In order from highest to lowest, the concentrations of analyte used were 25, 12.5, 6.25, 3.125 nM.

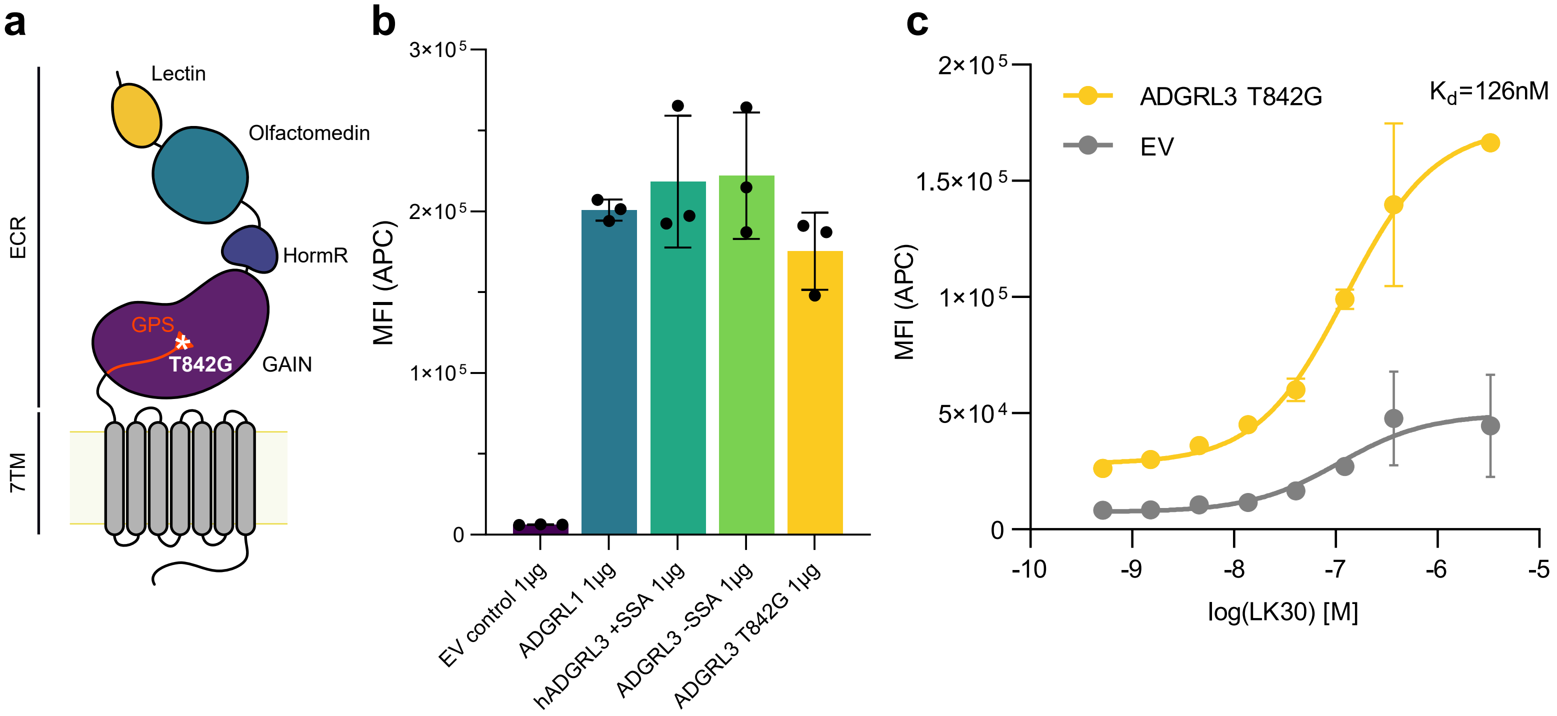

Supplementary Fig. 3: sAB LK30 binds to the ADGRL3 autoproteolysis mutant.

a Schematic diagram of full-length autoproteolysis mutant of ADGRL3. The Lectin domain is colored yellow, Olfactomedin - cyan, Hormone Binding Region - navy, GAIN - purple and 7TM - gray. The last β-strand of the GAIN domain is colored red and the autoproteolysis site within the GAIN domain (GPS) is presented as a triangle. Locaiton of single point mutation T842G in GPS that inhibits autoproteolysis shown as white star. **b** Cell surface expression of all constructs was tested by transfecting HEK293T cells with equal amount of construct DNA, and measuring the protein expression using anti-FLAG antibody by flow cytometry. Data are presented as mean ± SD of three repeats (n = 3) for a representative of three independent experiments. **c** Binding of LK30 to the ADGRL3 T842G autoproteolysis mutant was measured on HEK293T cells by flow cytometry. K_D value of LK30 binding was determined to be 126 nM. Data are presented as mean ± SD of three repeats (n = 3) for a representative of three independent experiments.

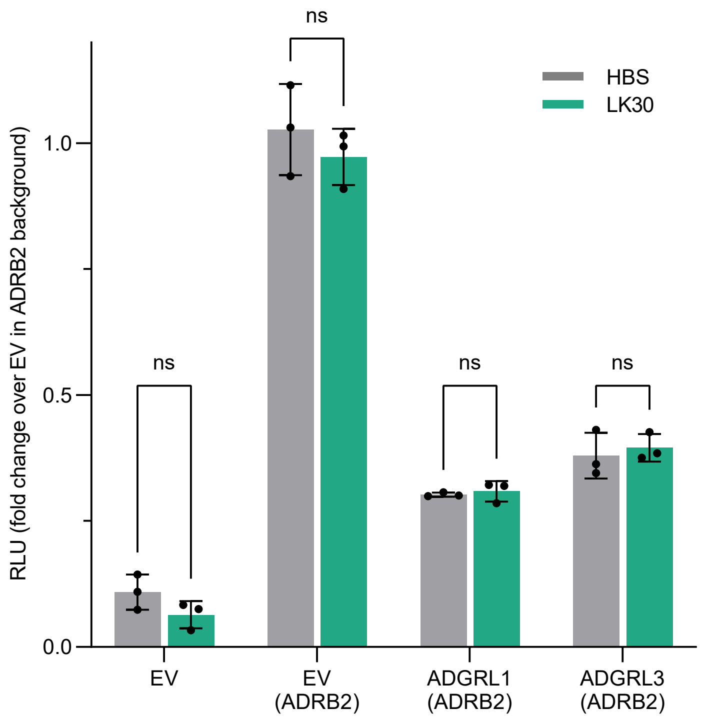

Supplementary Fig. 4:. LK30 does not affect ADGRL1 and ADGRL3 cAMP signaling.
ADGRL1 and ADGRL3 signaling measured by the cAMP assay in the presence of 2 µM of LK30. Data are shown as a fold change over EV in the ADRB2 background. RLU, relative luminescence units. Data are presented as mean ± SD of three repeats (n = 3) for a representative of three independent experiments, LK30 vs. HBS buffer treatment; two-way ANOVA. Source data are provided as a Source Data file.

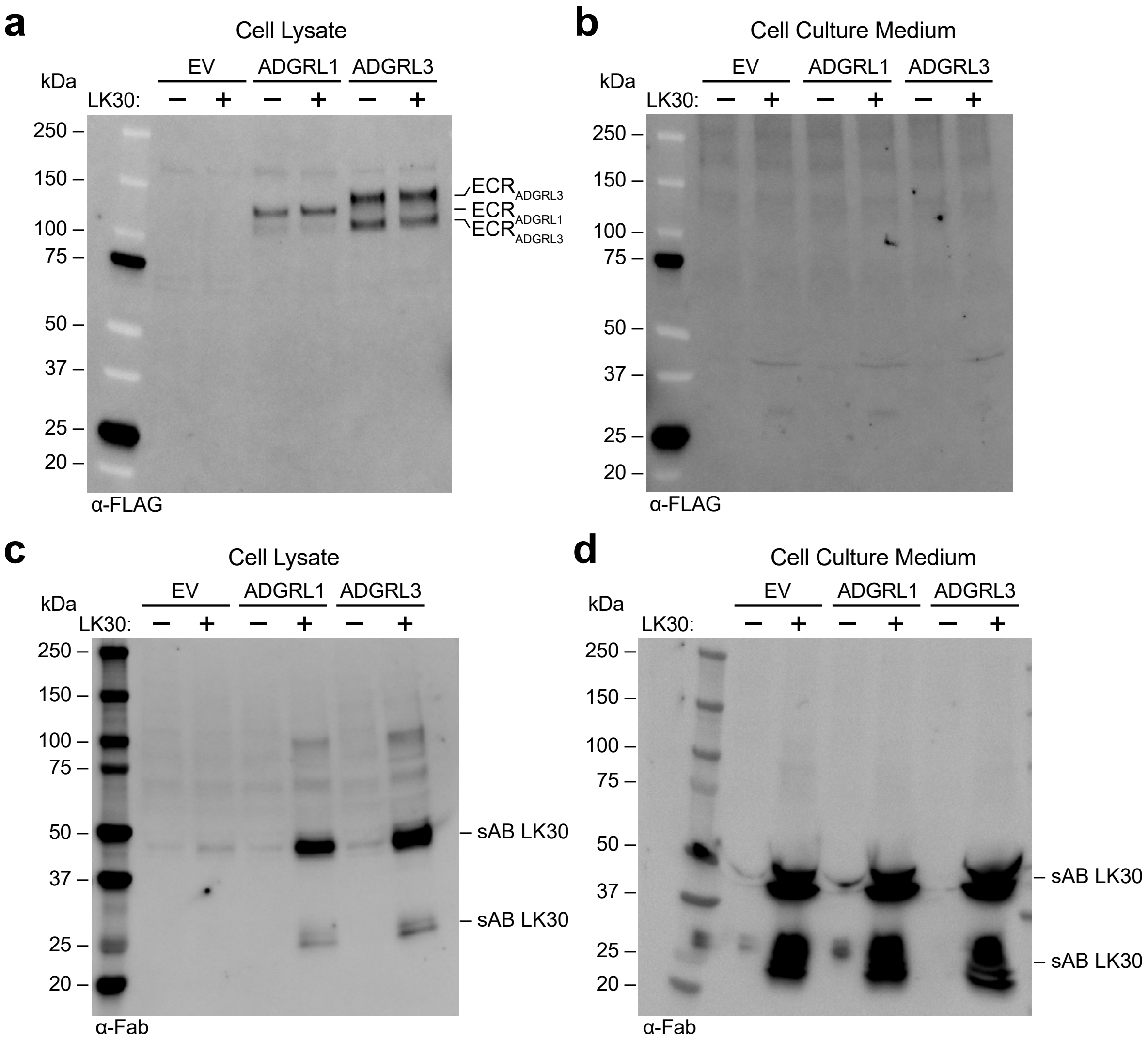

Supplementary Fig. 5: LK30 does not induce ECR dissociation of ADGRLs expressed on cell surface.

Western Blot analysis of Cell Lysates (**a** and **c**) and Culture Media (**b** and **d**) of HEK293T cell cultures transfected with empty vector, ADGRL1 or ADGRL3, with and without LK30 antibody treatement. Source data are provided as a Source Data file. Results representative of at least two independent experiments.

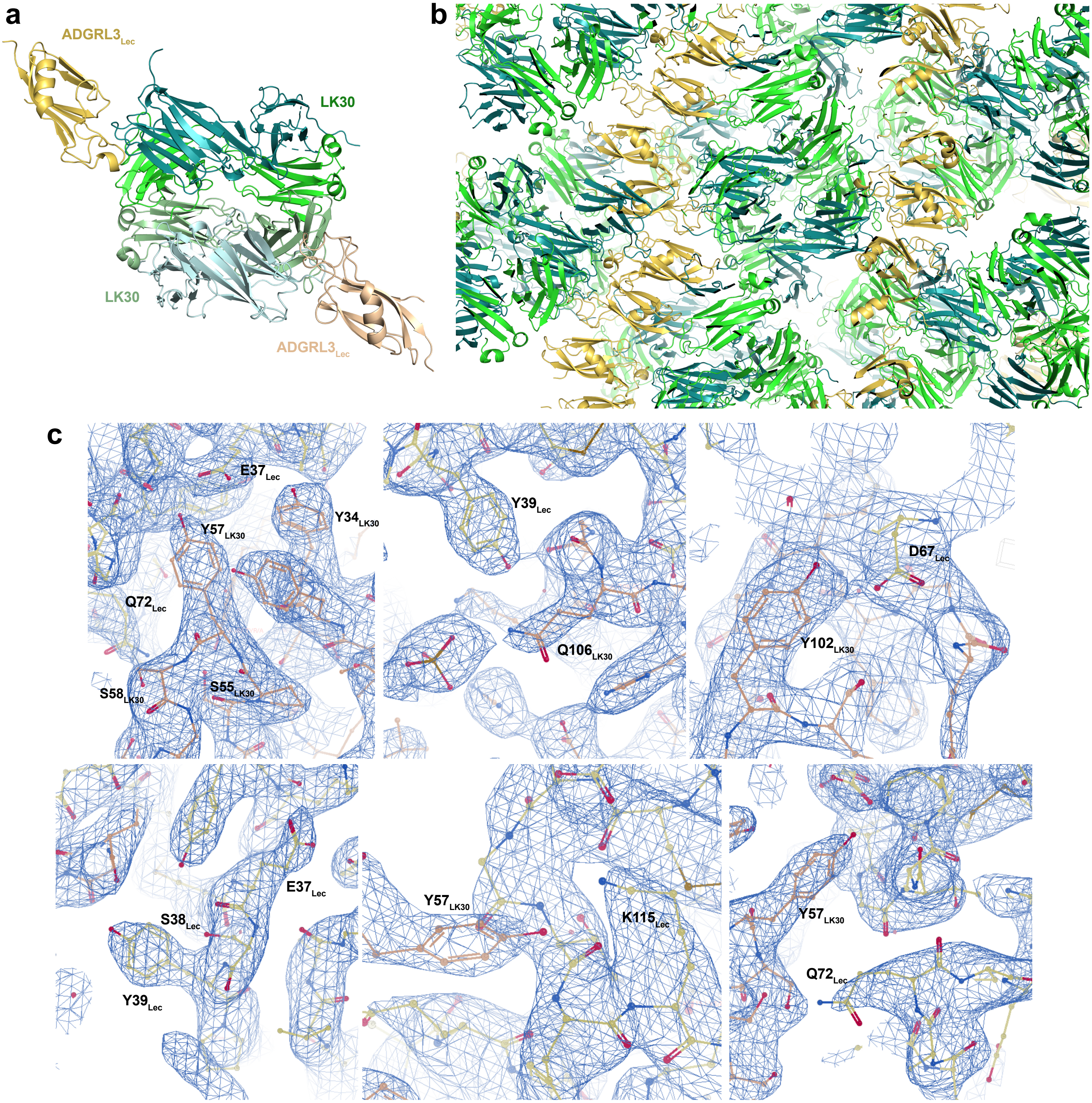

Supplementary Fig. 6: Crystal structure of ADGRL3/LK30 complex

a The structure of the complex was obtained in space group $P2_12_12_1$ with two $ADGRL3_{Lec}$/LK30 complexes in the asymmetric unit. **b** Analysis of the crystal packing shows the contacts in the crystal lattice are mediated primarily by the heavy chain (HC) and light chain (LC) of LK30 and Lec domain of ADGRL3. $ADGRL3_{Lec}$ is colored yellow while the HC and LC of LK30 are colored green. **c** Electron densities contoured at 1σ for the crucial residues at the $ADGRL3_{Lec}$/LK30 interface.

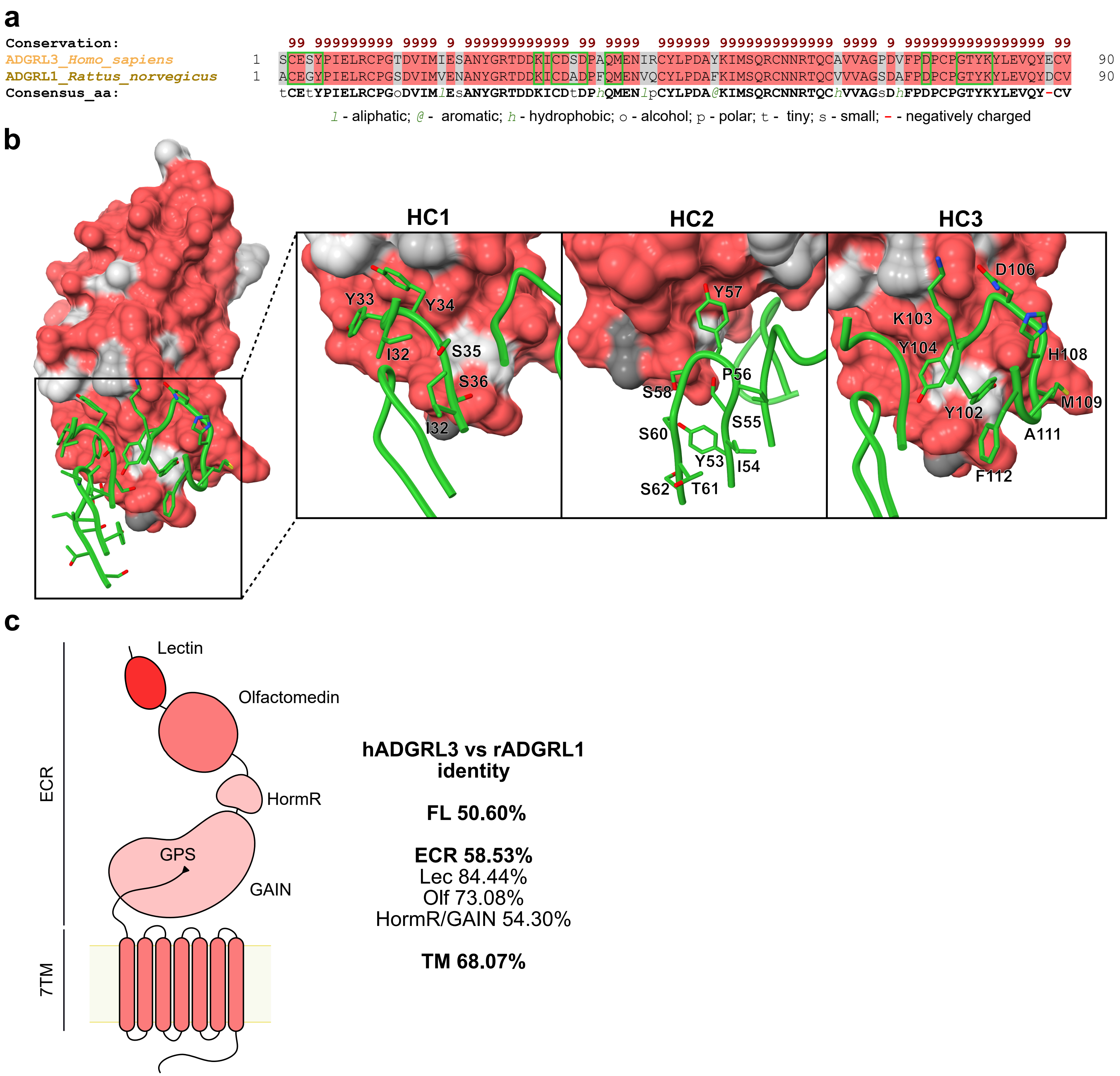

Supplementary Fig. 7: Conservation analysis of ADGRL3 and ADGRL1.
a Sequence alignment of Lectin domains of ADGRL3 and ADGRL1 by PROMALS 3D. Residues labeled in red are identical between ADGRL3 and ADGRL1, residues labeled in gray are semi-conserved. Semi-conserved residues are labeled below the alignment according to PROMALS 3D. **b** Surface conservation representation (gray, semi-conserved; red, identical) between the Lec domains of ADGRL3 and ADGRL1. The CDRs of the LK30 HC interacting with the Lec domain are shown in green, with detailed labels of each residue shown in the additional panels. **c** ADGRL scheme colored by sequence identity between domains of ADGRL1 and ADGRL3, with sequence identity reported.

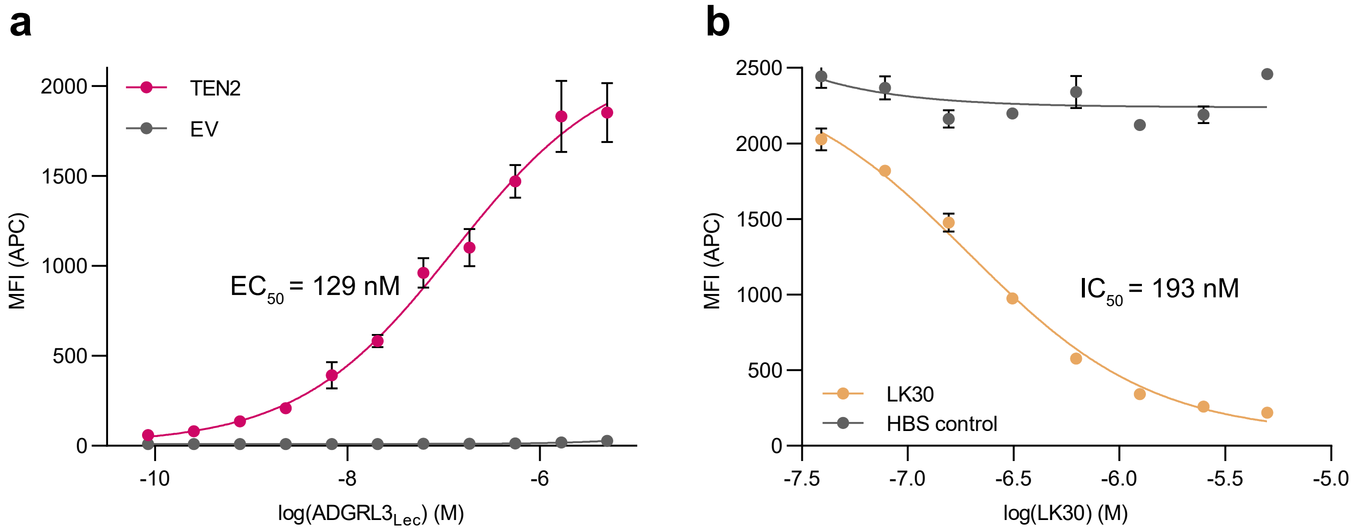

Supplementary Fig. 8: Characterization of ADGRL3 binding to TEN2.

a Binding activity of ADGRL3 Lec domain to TEN2 was measured using HEK293T cells expressing full-length TEN2 by flow cytometry. EC_{50} value of ADGRL3 Lec binding was determined as 129 nM, by fitting the data to the concentration-response curve in GraphPad Prism. Cells transfected with empty vector were used as negative control (gray curve). Data are presented as mean ± SD of three repeats (n = 3) for a representative of three independent experiments. **b** The inhibitory effect of LK30 binding to the TEN2/ADGRL3 complex was tested by addition of increasing concentrations of LK30 to pre-formed TEN2/ADGRL3 Lec complex on cell surface. IC_{50} value was determined as 193nM by fitting the data to the concentration-response curve in GraphPad Prism. HBS buffer addition to the pre-formed TEN2/ADGRL3 Lec was used as negative control (gray curve). Data are presented as mean ± SD of three repeats (n = 3) for a representative of three independent experiments. Source data are provided as a Source Data file.

a

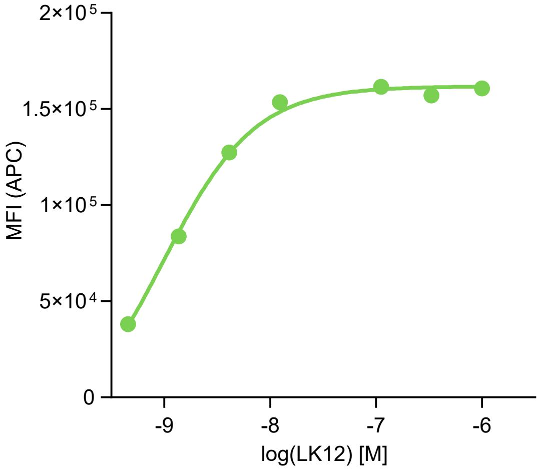

b

LK12	
**CDR**	**Sequence**
**L3**	FGYQLI
**H1**	VSSYSI
**H2**	SIYSYYGSTY
**H3**	EVYYFWEPYAYGF

Supplementary Fig. 9: Characterization of ADGRL3 binder sAB LK12.
a Binding activity of LK12 to the receptor was measured using HEK293T cells expressing full-length ADGRL3 by flow cytometry. **b** Sequences of LK12 CDRs.

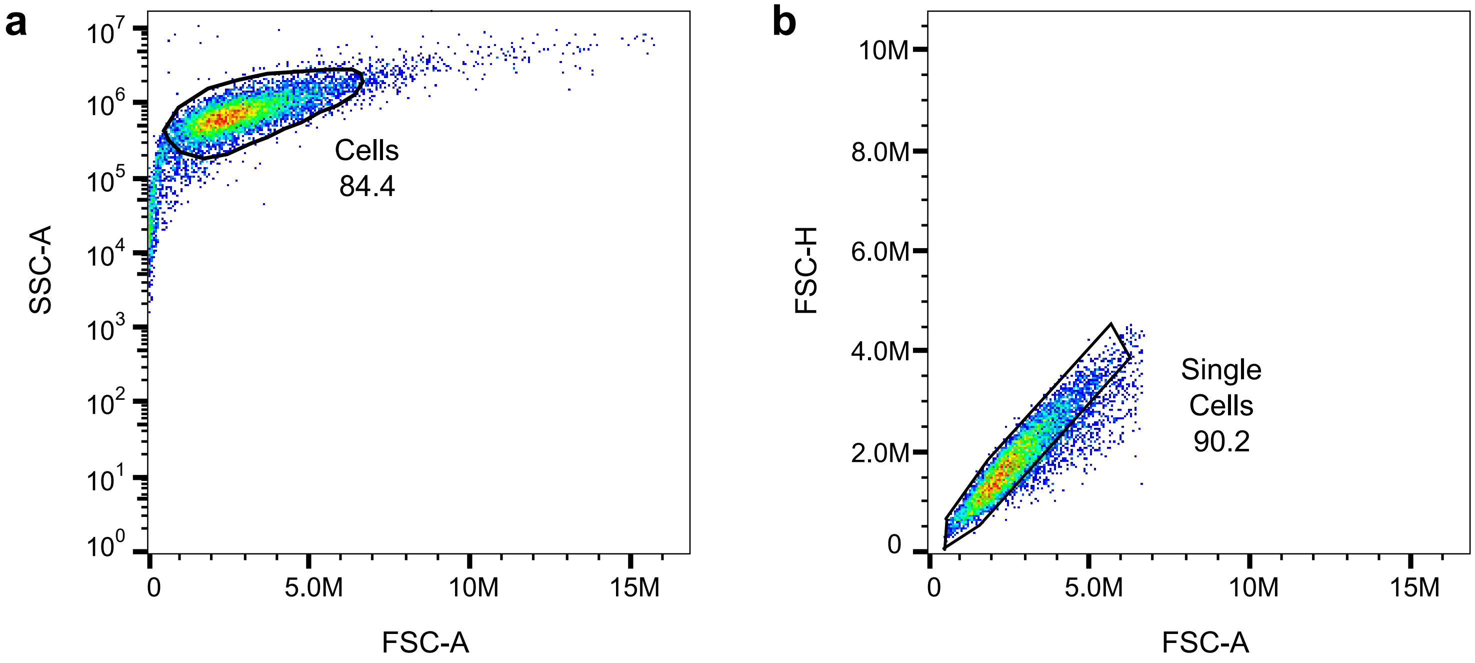

Supplementary Fig. 10: Gating strategy for flow cytometry experiments.
Two-step gating strategy was used for all flow cytometry data presented in this study: **a** Forward vs side scatter to separate cells from debris; **b** Height vs area forward scatter to select single cells. Gated cells were used to measure the mean fluorescent intensity (MFI) and MFI values were plotted in subsequent graphs. Presented an example for ADGRL1 cell surface expression experiment (Supplementary Fig. 3b).
