# Supplementary material for: Isoform- and ligand-specific modulation of the adhesion GPCR ADGRL3/Latrophilin3 by a synthetic binder
Source: Nat Commun. 2023 Feb 6;14:635. doi: 10.1038/s41467-023-36312-7 (PMC9902482; doi:10.1038/s41467-023-36312-7)
Supplement: Supplementary file 3 — Reporting Summary [file 41467_2023_36312_MOESM3_ESM.pdf]

nature portfolio

Corresponding author(s): Demet Arac

Last updated by author(s): Jan 17, 2023

Reporting Summary

Nature Portfolio wishes to improve the reproducibility of the work that we publish. This form provides structure for consistency and transparency in reporting. For further information on Nature Portfolio policies, see our Editorial Policies and the Editorial Policy Checklist.

Statistics

For all statistical analyses, confirm that the following items are present in the figure legend, table legend, main text, or Methods section.

n/a	Confirmed	
☐	☒	The exact sample size (*n*) for each experimental group/condition, given as a discrete number and unit of measurement
☐	☒	A statement on whether measurements were taken from distinct samples or whether the same sample was measured repeatedly
☐	☒	The statistical test(s) used AND whether they are one- or two-sided<br>*Only common tests should be described solely by name; describe more complex techniques in the Methods section.*
☒	☐	A description of all covariates tested
☒	☐	A description of any assumptions or corrections, such as tests of normality and adjustment for multiple comparisons
☐	☒	A full description of the statistical parameters including central tendency (e.g. means) or other basic estimates (e.g. regression coefficient) AND variation (e.g. standard deviation) or associated estimates of uncertainty (e.g. confidence intervals)
☐	☒	For null hypothesis testing, the test statistic (e.g. *F*, *t*, *r*) with confidence intervals, effect sizes, degrees of freedom and *P* value noted<br>*Give P values as exact values whenever suitable.*
☒	☐	For Bayesian analysis, information on the choice of priors and Markov chain Monte Carlo settings
☒	☐	For hierarchical and complex designs, identification of the appropriate level for tests and full reporting of outcomes
☒	☐	Estimates of effect sizes (e.g. Cohen's *d*, Pearson's *r*), indicating how they were calculated

Our web collection on statistics for biologists contains articles on many of the points above.

Software and code

Policy information about availability of computer code

Data collection	JBluIce-EPICS (x-ray diffraction collection), BioTek Gen5 (signaling data collection), Leica Fluorescent DMi8 LED Microscope
Data analysis	XDS (x-ray diffraction processing), PHENIX 1.19.2-4158 (x-ray structure determination), CCP4 7.1.016 (x-ray structure determination), GraphPad Prism 9.3.1 (signaling data analysis), ConSurf (structural conservation analysis), COOT 0.9.6 (Model building and refinement), PyMOL 2.4.0 & UCSF Chimera 1.11.2 (structure visualization), ImageJ 1.52e (image processing)

For manuscripts utilizing custom algorithms or software that are central to the research but not yet described in published literature, software must be made available to editors and reviewers. We strongly encourage code deposition in a community repository (e.g. GitHub). See the Nature Portfolio guidelines for submitting code & software for further information.

Data

Policy information about availability of data

All manuscripts must include a data availability statement. This statement should provide the following information, where applicable:

- Accession codes, unique identifiers, or web links for publicly available datasets
- A description of any restrictions on data availability
- For clinical datasets or third party data, please ensure that the statement adheres to our policy

The coordinates for the crystal structure of ADGRL3/LK30 generated in this study have been deposited in the Protein Data Bank [http://www.rcsb.org] under accession code PDB 8DJG [http://doi.org/10.2210/pdb8DJG/pdb]. All the other relevant structures referenced in this work are available under the accession codes 6VHH [http://doi.org/10.2210/pdb6VHH/pdb], 4XWO [http://doi.org/10.2210/pdb4XWO/pdb], 5AFB [http://doi.org/10.2210/pdb5AFB/pdb], 5UCB [http://doi.org/10.2210/pdb5UCB/pdb], 6SKA [http://doi.org/10.2210/pdb6SKA/pdb] and 5FTU [http://doi.org/10.2210/pdb5FTU/pdb]. The authors declare that all data supporting the findings of this study are available within the article and the source data underlying Fig. 1B, 1G, 2A-C, 5E, 5J and Supplementary Figs. 4, 5 and 8 are provided as a Source Data file. The gating strategy for flow cytometry experiments can be found in Supplementary Fig. 10.

Human research participants

Policy information about studies involving human research participants and Sex and Gender in Research.

Reporting on sex and gender	N/A
Population characteristics	N/A
Recruitment	N/A
Ethics oversight	N/A

Note that full information on the approval of the study protocol must also be provided in the manuscript.

Field-specific reporting

Please select the one below that is the best fit for your research. If you are not sure, read the appropriate sections before making your selection.

☒ Life sciences ☐ Behavioural & social sciences ☐ Ecological, evolutionary & environmental sciences

For a reference copy of the document with all sections, see nature.com/documents/nr-reporting-summary-flat.pdf

Life sciences study design

All studies must disclose on these points even when the disclosure is negative.

Sample size	No sample size calculation was performed. A sample size of n=3 is commonly used in biological studies using cell cultures. All experiments were performed in at least three independent culture preparations with at least three independent biologial replicates.
Data exclusions	No data were excluded.
Replication	Signaling assays were done in triplicate, and all attempts at replication were successful. Image analysis were done in three independent replicates with quantifications from randomly selected imaging fields per replicate. All experiments were repeated in part or in whole at least twice to ensure reproducibility.
Randomization	Cells placed in different positions on 96-well signaling assay plate and randomly allocated into control and treatment groups. Quantifications from randomly selected imaging fields per experimental condition replicate.
Blinding	Blinding is not relevant to this study, all experiments were performed based on standardized protocols and readouts and are not influenced by the investigator.

Reporting for specific materials, systems and methods

We require information from authors about some types of materials, experimental systems and methods used in many studies. Here, indicate whether each material, system or method listed is relevant to your study. If you are not sure if a list item applies to your research, read the appropriate section before selecting a response.

Materials & experimental systems

n/a	Involved in the study
☐	☒ Antibodies
☐	☒ Eukaryotic cell lines
☒	☐ Palaeontology and archaeology
☒	☐ Animals and other organisms
☒	☐ Clinical data
☒	☐ Dual use research of concern

Methods

n/a	Involved in the study
☒	☐ ChIP-seq
☐	☒ Flow cytometry
☒	☐ MRI-based neuroimaging

Antibodies

Antibodies used

HRP-conjugated Mouse Anti-M13 monoclonal antibody (GE Healthcare,

HRP-conjugated Goat Anti-Human F(ab')$_2$ fragment specific antibody (Jackson ImmunoResearch, 109-035-006)
AlexaFluor647-conjugated Goat Anti-Human IgG F(ab')$_2$ fragment specific antibody (Jackson ImmunoResearch, 109-605-006)
Mouse Anti-FLAG antibody (Sigma-Aldrich, F3165)
AlexaFluor488-conjugated Donkey Anti-Mouse antibody (Invitrogen, A21202)
THE™ DYKDDDDK Tag Antibody [iFluor 488], mAb, Mouse (Genescript, A01809)

Validation

Most commercially available antibodies (from various suppliers) that are being used during the course of our studies have been validated by our lab and others through comparison of obtained immunohistochemistry (IHC) and/or Western blot (WB) results between labs and, in many cases, confirmation of specificity using mutant mice lacking the proteins detected by the antibodies. We validate each new lot of antibody by reproducing results obtained with previous batches of antibody.

(from Sigma-Aldrich website) Anti Flag M2 antibody is used for the detection of Flag fusion proteins.This monoclonal antibody is produced in mouse and recognizes the FLAG sequence at the N-terminus, Met N-terminus, and C-terminus. The antibody is also able to recognize FLAG at an internal site. M2, unlike M1 antibody is not Calcium dependent. Antibody is recommended for use in immunoblotting, immunoprecipitation, immunocytochemistry, immunofluorescence, ELISA, electron microscopy, flow cytometry and supershift assays.

Eukaryotic cell lines

Policy information about cell lines and Sex and Gender in Research

Cell line source(s)

ATCC: HEK293T, HEK293
Thermo Fisher: Sf9, High Five

Authentication

HEK293T/HEK293 cell lines (from ATCC) have been validated by the supplier through STR analysis and cytogenetic studies. Sf9 and High Five were not authenticated. We also employ a protocol for sequencing of baculoviral stocks postamplification to confirm identities of proteins over-expressed in lepidopteran insect cells, especially for mutant variants that are hard to differentiate otherwise.

Mycoplasma contamination

Cell lines tested negative for mycoplasma contamination (ATCC)

Commonly misidentified lines
(See ICLAC register)

No commonly misidentified cell lines were used in this study

Flow Cytometry

Plots

Confirm that:

☒ The axis labels state the marker and fluorochrome used (e.g. CD4-FITC).

☒ The axis scales are clearly visible. Include numbers along axes only for bottom left plot of group (a 'group' is an analysis of identical markers).

☒ All plots are contour plots with outliers or pseudocolor plots.

☒ A numerical value for number of cells or percentage (with statistics) is provided.

Methodology

Sample preparation

Samples are transiently transfected HEK293 cells. Flow cytometry was used to measure cell surface expression of transiently expressed proteins and the binding between ADGRLs and LK30.

Instrument

BD Accuri C6

Software	FlowJo
Cell population abundance	No sorting was performed.
Gating strategy	Two-step gating strategy was used for all flow cytometry data presented in this study. Forward vs side scatter to separate cells from debris and height vs area forward scatter to select single cells.

☒ Tick this box to confirm that a figure exemplifying the gating strategy is provided in the Supplementary Information.
